# Pemphigus Vulgaris in Old Patient

**DOI:** 10.1155/2021/3946161

**Published:** 2021-08-23

**Authors:** Bassel Tarakji

**Affiliations:** Department of Oral and Maxillofacial Surgery and Diagnostic Sciences, College of Dentistry Prince Sattam Bin Abdulaziz University, Al-Kharj, Saudi Arabia

## Abstract

Pemphigus vulgaris (PV) is a chronic, autoimmune, intraepidermal blistering disease of the skin and mucous membranes. The first clinical manifestation is often the development of intraoral lesions, and later, the lesions involve the other mucous membranes and skin. The etiological factors of this disease still remain unknown, although the presence of autoantibodies is consistent with an autoimmune disease. A 73-year-old man had bullous lesions on gingiva, oral mucosa first, then scalp, trunk, and face. An oral medicine specialist suspects the lesion in differential diagnosis in the first presentation of oral lesions and follows up the patient, and then these bullous lesions presented on the skin. In this article, a patient had received oral prednisolone (80 mg/kg/day) and azathioprine, then tapered oral prednisolone to 40 mg/day, with a reduction of 5 mg/day every three weeks. The patient shows remission of these lesions, and complication of this treatment includes osteoporosis, hyperglycemia, and hypertension.

## 1. Introduction

Pemphigus vulgaris (PV) is an autoimmune vesiculobullous skin disease in that the immune system produces autoantibody against the specific proteins of the desmosomal adhesion complex that led to the intraepidermal blister formation [1]. Pemphigus vulgaris is seen between third and sixth decades of life [2]. Blisters are associated with the binding of IgG autoantibodies of keratinocyte cell surface molecules [1, 2]. PV antibodies bind to keratinocyte desmosomes and to desmosome-free areas of the keratinocyte cell membrane [3]. Pemphigus vulgaris is slightly predominate in women and manifests in adults during fifth and sixth ages of life [3]. This case is very rare in Syria because most cases identified in literature are middle-aged, not elderly [3]. Most patients complain of painful mucous membrane erosions and may be the only sign of PV of average few months before skin lesions develop [3, 4]. Lesions may occur anywhere on the oral mucosa [4, 5], but the buccal mucosa is the most commonly affected site [4, 5]. Erosive or desquamative gingivitis is a common manifestation of the disease [4–6]. Early oral lesions of PV are difficult to diagnose, and dentists and oral medicine specialists have the main role in early detection.

## 2. Case Presentation

A 73-year-old male patient from Aleppo city in Syria referred to a dermatology private clinic and oral medicine clinic in Aleppo University.

**Figure 1:**
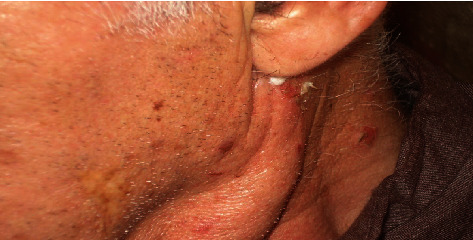
Bullous blisters on the face.

**Figure 2:**
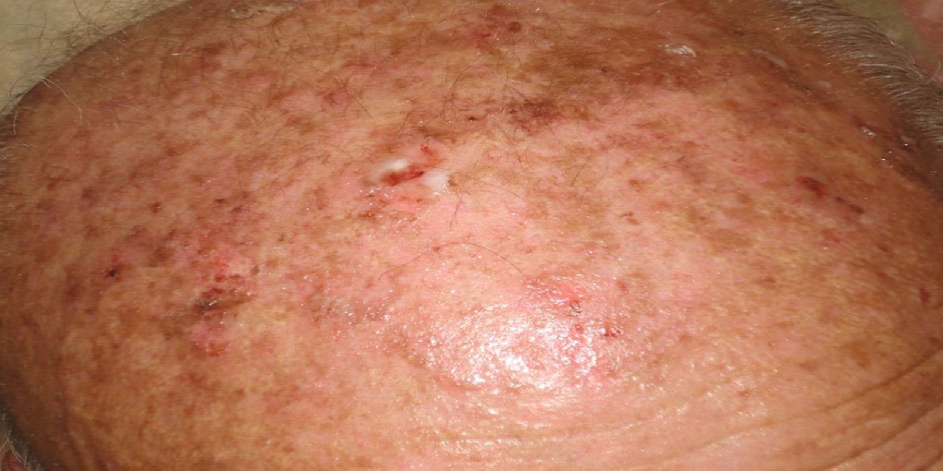
Bullous blisters on the scalp.

**Figure 3:**
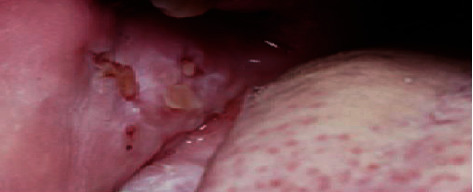
Erosions on the buccal mucosa.

**Figure 4:**
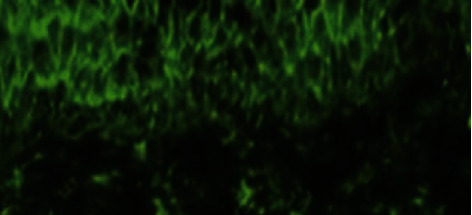
Immunofluorescence showed deposition of IgG in the epidermis.

The patient complains of bullous lesions on gingiva, oral mucosa first, then on scalp, trunk, and face (Figures 1–3). The patient indicates no history of medication. I have followed up the patient to detect bullous lesions if it develops on the skin. Crusted plaques were identified on the scalp, trunk, and face. In oral medicine clinic, clinical intraoral examination indicated to erosions and erythematous on gingiva and buccal mucosa. The patient indicated in the medical history that the oral erosion lesions started few months before the presentation of bullous lesions on his skin. I have referred the patient to a dermatology clinic for evaluation as well. Clinical examination of his skin indicates that the hyperpigmented circular patches were identified over the chest and abdomen as well as a sequel of rupture of bullae. Biopsy of skin lesions from fresh vesicles showed a suprabasal cleft formation and a row of “tomb-stone” appearance of basal cells. Direct immunofluorescence showed deposition of IgG in epidermis (Figure 4). The diagnosis confirmed as pemphigus vulgaris (Table 1).

The patient received oral prednisolone (80 mg/kg/day) and azathioprine. The consent form was signed by the patient, and ethical approval was taken from Aleppo University. The dose of prednisolone was slowly tapered down to 40 mg/day (then decrease to 5 mg every three weeks). Specialists in dermatology and oral medicine follow the lesions on his skin and mouth, respectively. All lesions show complete recovery. The patient had received the high doses of prednisolone in hospital at the beginning of treatment. The daily evaluation for this case by specialists in dermatology, oral medicine, and internal medicine is very strict. The patient has complication such as diabetic, fasting blood sugar showing 160 mg/dL, depression, and osteoporosis. The patient remained under the treatment of 5 mg/day and 100 mg/day of prednisolone and azathioprine, respectively. The patient shows complete remission achieved three years from diagnosis. The patient shows side effects such as osteoporosis hyperglycemia and hypertension.

## 3. Discussion

I have indicated in this case that a 73-year-old patient with pemphigus vulgaris, complaining of mucocutaneous lesions, has fully recovered from the lesions. This case is consistent with Patvekar and Sadana [6] who reported a 94-year-old woman diagnosed with pemphigus vulgaris. Langan et al. [7] have reported that the median age at presentation in the UK is 71 years old (range, 21-102). Martel et al. [8] reported that PV incidence occurs in the third and sixth decades of life. PV occurs most often at the fifth decade of life and not in the elderly [9, 10]. Although the development of blisters and erosions in elderly patients is not fully understood, but this could be of immunologic dysregulation which occurs with aging. Also, it might be that there are differences in the skin between young and elderly patients. Parker and Kelfresh [11] indicated that the development of early blisters is more readily in the elderly skin compared to young patients. Pemphigus vulgaris is a high-risk disease and needs urgent treatment, and its prognosis is not good if the patient left without treatment. Treatment includes corticosteroids and/or immunosuppressor medication such as azathioprine and methotrexate. The patient received the initial low-dose oral prednisolone at the concentration of 80 mg/day for two weeks while he was hospitalized. The prednisolone dose was reduced to 40 mg/day. The reason for this decrease was to minimize the risk of potential side effects [2]. Based on the clinical examinations and laboratory tests, the physicians decided to reduce the dose of prednisolone to 40 mg/day, then reduced to 5 mg/day every three weeks as they evaluate the patient daily, most blisters and erosions show good improvement, and the general health of the patient was good. Herman et al. [2] have suggested a reduction of 5 to 10 mg of prednisolone per week and more slowly less than 20 mg prednisolone per day; however, the dosing schedules are experimental and can modify it depending on the practical experience and patients' situation [2]. The treatment may be withdrawn if there is prolonged clinical healing. The use of high doses of systemic corticosteroids is associated with complication such as osteoporosis and hyperglycemia [12–14]. In this case, the patient shows osteoporosis due to the aging and long use of prednisolone. Also, my patient shows hyperglycemia due to a high dosage of corticosteroids, but his blood sugar before start treatment was borderline, 125 mg/dL. The patient had hypertension due to depression and long-term treatment for pemphigus vulgaris.

This complicated case needs strict joint consultation to find the treatment plan. The role of oral medicine physician is so important to detect the early lesions in the mouth in cooperation with a dermatologist and internal medicine to follow up the patient. I believe that dentists and especially oral medicine can evaluate the abnormal lesions related to autoimmunity disease, and that can help the patient to detect these lesions as early as possible. Assessment of pemphigus vulgaris in literature especially in elderly patients is so limited.

It is recommended to highlight the difficulties in this complicated disease especially in elderly patients over 70 years old. I conclude that the disease has subsided in my case due to the age-related immune suppression.

## Figures and Tables

**Table 1:** The description of this case.

Main variables	Description
Age	73
Gender	Male
Patient history	No medication
Clinical features and oral examination	Bullous lesions on his head, trunk, and face; erosions on buccal mucosa, erythematous areas on the gingiva (desquamative gingivitis); and oral mucosa
Diagnosis	Pemphigus vulgaris
Treatment	80 mg/d oral prednisolone, then reduced to 40 mg/day, and subsequently a 5 mg reduction of the prednisolone every three weeks to complete the remission of the lesion. Patient remained under treatment of 5 mg/d prednisolone and 100 mg/d azathioprine

## References

[1] Grando S. A. (2012). Pemphigus autoimmunity: hypotheses and realities. *Autoimmunity*.

[2] Harman K. E., Albert S., Black M. M. (2003). Guidelines. *The British Journal of Dermatology*.

[3] Ali A., AliReza Y., Gita F. (2006). Pemphigus vulgaris in Iran: epidemiology and clinical profile. *SKINmed*.

[4] Dagistan S., Goregen M., Miloglu O., Çakur B. (2008). Oral pemphigus vulgaris: a case report with review of the literature. *Journal of Oral Science*.

[5] Karagir A., Ranpise S. G., Ranpise T. (2012). Oral pemhigus vulgaris-a case report. *JIDA*.

[6] Patvekar M. A., Sadana D. (2014). Pemphigus vulgaris in an elderly patient. *Indian Journal of Dermatology*.

[7] Langan S. M., Smeeth L., Hubbard R., Fleming K. M., Smith C. J. P., West J. (2008). Bullous pemphigoid and pemphigus vulgaris--incidence and mortality in the UK: population based cohort study. *BMJ*.

[8] Martel P., Cordel N., Courville P., Gilbert D., Musette P., Joly P. (2002). Pemphigus with clinical, histological and immunological features of both vulgaris and foliaceus subtypes. *The British Journal of Dermatology*.

[9] Ikeda S., Imamura S., Hashimoto I., Morioka S., Sakuma M., Ogawa H. (2003). History of the establishment and revision of diagnostic criteria, severity index and therapeutic guidelines for pemphigus in Japan. *Archives of Dermatological Research*.

[10] Shamim T., Varghese V. I., Shameena P. M., Sudha S. (2008). Pemphigus vulgaris in oral cavity: clinical analysis of 71 cases. *Medicina Oral, Patología Oral y Cirugía Bucal*.

[11] Parker S. R., MacKelfresh J. (2011). Autoimmune blistering diseases in the elderly. *Clinics in Dermatology*.

[12] Kasperkiewicz M., Schmidt E., Zillikens D. (2012). Current therapy of the pemphigus group. *Clinics in Dermatology*.

[13] Ljubojevic S., Lipozencic J., Brenner S., Budimcic D. (2002). Pemphigus vulgaris: a review of treatment over a 19-year period. *Journal of the European Academy of Dermatology and Venereology*.

[14] Chovatiya R., Silverberg J. I. (2020). Association of pemphigus and pemphigoid with osteoporosis and pathological fractures. *Archives of Dermatological Research*.

